# SLC6A14 and SLC38A5 Drive the Glutaminolysis and Serine–Glycine–One-Carbon Pathways in Cancer

**DOI:** 10.3390/ph14030216

**Published:** 2021-03-04

**Authors:** Tyler Sniegowski, Ksenija Korac, Yangzom D. Bhutia, Vadivel Ganapathy

**Affiliations:** Department of Cell Biology and Biochemistry, Texas Tech University Health Sciences Center, Lubbock, TX 79430, USA; Tyler.Sniegowski@ttuhsc.edu (T.S.); ksenija.korac@ttuhsc.edu (K.K.); yangzom.d.bhutia@ttuhsc.edu (Y.D.B.)

**Keywords:** cancer-specific metabolism, one-carbon metabolism, glutamine addiction, oncometabolites, amino acid transporters, SLC6A14 and SLC38A5

## Abstract

The glutaminolysis and serine–glycine–one-carbon pathways represent metabolic reactions that are reprogramed and upregulated in cancer; these pathways are involved in supporting the growth and proliferation of cancer cells. Glutaminolysis participates in the production of lactate, an oncometabolite, and also in anabolic reactions leading to the synthesis of fatty acids and cholesterol. The serine–glycine–one-carbon pathway is involved in the synthesis of purines and pyrimidines and the control of the epigenetic signature (DNA methylation, histone methylation) in cancer cells. Methionine is obligatory for most of the methyl-transfer reactions in the form of S-adenosylmethionine; here, too, the serine–glycine–one-carbon pathway is necessary for the resynthesis of methionine following the methyl-transfer reaction. Glutamine, serine, glycine, and methionine are obligatory to fuel these metabolic pathways. The first three amino acids can be synthesized endogenously to some extent, but the need for these amino acids in cancer cells is so high that they also have to be acquired from extracellular sources. Methionine is an essential amino acid, thus making it necessary for cancer cells to acquire this amino acid solely from the extracellular milieu. Cancer cells upregulate specific amino acid transporters to meet this increased demand for these four amino acids. SLC6A14 and SLC38A5 are the two transporters that are upregulated in a variety of cancers to mediate the influx of glutamine, serine, glycine, and methionine into cancer cells. SLC6A14 is a Na^+^/Cl^−^ -coupled transporter for multiple amino acids, including these four amino acids. In contrast, SLC38A5 is a Na^+^-coupled transporter with rather restricted specificity towards glutamine, serine, glycine, and methionine. Both transporters exhibit unique functional features that are ideal for the rapid proliferation of cancer cells. As such, these two amino acid transporters play a critical role in promoting the survival and growth of cancer cells and hence represent novel, hitherto largely unexplored, targets for cancer therapy.

## 1. Introduction

Cancer cells have been shown to hijack specific metabolic pathways that occur in normal cells and reprogram them to meet the increased demands for various nutrients and metabolites in support of their rapid proliferation. The altered metabolism encompasses all three major nutrient groups: sugars, amino acids, lipids. This new understanding of metabolic reprogramming in cancer has identified novel drug targets for cancer therapy, thus ushering the area of cancer therapeutics into new territories. In this review, we focus on cancer-cell-specific pathways associated with glucose and amino acid metabolism, with a special emphasis on the amino acids glutamine, serine, glycine, and methionine and the two amino acid transporters SLC6A14 and SLC38A5 that supply the cancer cells with these amino acids.

## 2. Metabolic Reprogramming in Cancer Cells

Cancer cells must acquire certain specific biochemical and cell-biological features to ensure their characteristic sustained rapid growth and proliferation. This includes, but is not limited to, the anabolic capacity to synthesize lipids (phospholipids, cholesterol) necessary for membrane biogenesis, and macromolecules (nucleic acids, proteins) necessary for cell division, robust antioxidant machinery to prevent detrimental effects of oxidative stress, fine-tuning of regulatory pathways to balance the biological needs and potential deleterious effects of excess in case of specific nutrients (e.g., iron), generation of specific metabolites with unique functions suitable to sustain cell survival (oncometabolites), and appropriate changes in epigenetic control of gene transcription to promote the proteomic/enzymatic profile optimal for rapid growth with simultaneous suppression of senescence and cell death. Cancer cells achieve these features by judiciously reprogramming metabolic pathways via suppression, activation, or modification of specific biochemical reactions. None of these processes is feasible without the substrates to feed into these reactions, and cancer cells need to either generate these substrates endogenously or obtain them from the extracellular milieu. Endogenous synthesis is accomplished by the reprogrammed metabolic pathways, whereas acquisition from extracellular sources is made through the upregulation of selective transporters in the plasma membrane. The major nutrients that are imported from outside to support cancer growth include glucose, amino acids, vitamins, and minerals. The resultant increased availability of substrates from endogenous and exogenous sources fuels the metabolic pathways, such as aerobic glycolysis, glutaminolysis, reductive carboxylation, serine–glycine–one-carbon pathway, synthesis of oncometabolites, and control of epigenetic landscape; there have been numerous outstanding reviews in recent years focusing on these specific pathways and their relevance to cancer [1,2,3,4,5]. None of these pathways is necessarily exclusive to cancer cells. These pathways do occur in normal cells depending on specific biologic conditions of the cells; cancer cells hijack these pathways and reprogram them to best suit their needs.

## 3. Aerobic Glycolysis

**Figure 1:**
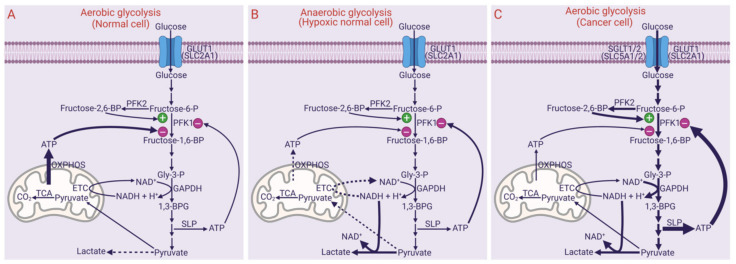
Differential features of aerobic glycolysis in normal cells (**A**), anaerobic glycolysis in normal cells (**B**), and aerobic glycolysis in cancer cells (**C**). PFK1, phosphofructokinase-1; PFK2, phosphofructokinase-2; Gly-3-P, glyceraldehyde-3-phosphate; 1,3-BPG, 1,3-bisphosphoglycerate; TCA, tricarboxylic acid cycle; ETC, electron transport chain; OXPHOS, oxidative phosphorylation wherein phosphorylation of ADP is coupled to oxidation of NADH and FADH_2_, thus generating ATP; SLP, substrate-level phosphorylation wherein phosphorylation of ADP is coupled to the hydrolysis of high-energy substrates 1,3-bisphosphoglycerate and phosphoenolpyruvate, thus generating ATP; GLUT1, glucose transporter 1; SGLT, sodium-coupled glucose transporter; SLC, solute carrier.

Aerobic glycolysis, also widely known as the Warburg effect, is a process in which glucose is metabolized with lactic acid as the end product in the presence of sufficient oxygen. This is similar to the glycolytic pathway that occurs in mature erythrocytes and a variation of the glycolytic pathway that occurs in skeletal muscle and other cell types in the absence of sufficient oxygen. In erythrocytes, the absence of mitochondria prevents the oxidation of pyruvate generated at the end of glycolysis despite the presence of oxygen. In other cells that have intact mitochondria, hypoxia prevents the oxidation of pyruvate. When glucose gets converted to pyruvate, Nicotinamide adenine dinucleotide (NAD^+^) is consumed at the reaction mediated by glyceraldehyde-3-phosphate dehydrogenase (glyceraldehyde-3-phosphate + Pi + NAD^+^ → 1,3-bisphosphoglycerate + NADH + H^+^). Because NAD^+^ is a coenzyme present in limited quantities, glycolysis cannot continue unless it is regenerated from the oxidation of NADH. This regeneration occurs in cells with mitochondria in the presence of sufficient oxygen when NADH goes through the electron transport chain and oxidative phosphorylation where electrons in NADH are transferred to molecular oxygen to produce water and NAD^+^ is regenerated in the process. However, ATP (adenosine trisphosphate) produced in oxidative phosphorylation negatively controls glycolysis by allosterically inhibiting the critical rate-limiting enzyme phosphofructokinase-1. Thus, glycolysis occurs at a relatively slower rate in the presence of sufficient oxygen in cells with intact mitochondria, but maximal energy is derived by oxidizing glucose completely into CO_2_. Accordingly, most of ATP generated in normal cells in the presence of oxygen (i.e., aerobic glycolysis in normal cells) arises from oxidative phosphorylation in mitochondria; ATP generated via substrate-level phosphorylation in the glycolytic pathway in the cytoplasm contributes relatively much less to total ATP (Figure 1A). Under hypoxic conditions as occurs in exercising muscle or ischemic heart, the electron transport chain and oxidative phosphorylation operate ineffectively, thus resulting in reduced NADH oxidation and ATP production within the mitochondria. The ineffective regeneration of NAD^+^ should stop glycolysis at the level of glyceraldehyde-3-phosphate dehydrogenase, but paradoxically glycolysis continues to operate under hypoxic conditions by two mechanisms: (i) reduced ATP, which relieves the inhibition of phosphofructokinase-1, and (ii) regeneration of NAD^+^ from NADH independent of the electron transport chain, which occurs via conversion of pyruvate to lactate mediated by lactate dehydrogenase (pyruvate + NADH + H^+^ → lactate + NAD^+^). Because glycolysis continues under hypoxic conditions in this scenario, this process is called “anaerobic glycolysis”, where the end product is lactic acid. Accordingly, hypoxic cells generate relatively less ATP, and most of it comes from substrate-level phosphorylation in the glycolytic pathway in the cytoplasm (Figure 1B). In mature erythrocytes, the absence of mitochondria prevents regeneration of NAD^+^ from NADH via electron transport chain; consequently, glycolysis in these cells relies on the conversion of pyruvate to lactate as the only means to regenerate NAD^+^ to sustain glycolysis. As a result, lactic acid is the end product of glycolysis in erythrocytes despite the presence of oxygen. 

In cancer cells in vivo, glycolysis results in the production of lactic acid. This is largely due to the fact that cancer cells at the center of solid tumors are relatively hypoxic because of their location far from the blood vessels (the rate of cancer cell proliferation outpaces the rate of vasculogenesis). If this were to be the sole reason for lactic acid being the end product of glycolysis in cancer cells, it is no different from what happens in normal cells under hypoxia; then, it ought to be called “anaerobic glycolysis”. Interestingly, this is not the whole picture in cancer cells. When cancer cells are cultured in vitro, even in the presence of normal levels of oxygen, glycolysis produces lactic acid. This cannot be explained without invoking some specific metabolic reprogramming in cancer cells. Irrespective of what underlies this unique phenomenon, lactic acid is the end product of glycolysis in cancer cells even in the presence of normal oxygen. This led to the coining of the term “aerobic glycolysis” to differentiate glycolysis in cancer cells from the “anaerobic glycolysis” that occurs in normal cells under hypoxic conditions. “Aerobic glycolysis” in cancer cells is still different from aerobic glycolysis in normal cells because glucose is converted predominantly into lactic acid in the former case, whereas glucose gets converted into CO_2_ in the latter case. This does not mean that cancer cells are deficient in ATP; in fact, cancer cells must produce more than normal ATP to support their rapid proliferation and growth. There are possibly several mechanisms for accelerated glycolysis with lactic acid as the end product in cancer cells even in the presence of oxygen: (i) mitochondrial respiration is suppressed to reduce the production of reactive oxygen species, which would otherwise be detrimental to cancer cells, thus necessitating accelerated glycolysis to produce more ATP via substrate-level phosphorylation to compensate for the decrease in ATP generation in mitochondria via oxidative phosphorylation [6]; (ii) suppression of lactate dehydrogenase-B (LDH-B) expression by epigenetic regulation and the upregulation of LDH-A by oncogene (c-Myc)- and HIF-1α (hypoxia-inducible factor-1α)-dependent mechanisms, with the resultant increase in LDH-A/LDH-B ratio favoring the conversion of pyruvate to lactate [7]; (iii) increased NAD^+^ regeneration from NADH by LDH-A, thus compensating for the decrease in mitochondria-dependent NAD^+^ regeneration to maintain the rate of glycolysis; (iv) inhibition of prolyl hydroxylase by lactate, thereby causing an increase in HIF-1α even in the presence of oxygen via protection from proteasomal degradation [6]; (v) c-Myc-mediated increase in the levels of fructose-2,6-bisphosphate, an allosteric activator of phoshofructokinase-1 that relieves the inhibition by ATP [8]; (vi) HIF-1α-mediated induction of the H^+^-coupled monocarboxylate transporter 4 (MCT4/SLC16A3) to export excess lactate out of the cancer cells to avoid intracellular acidification and associated detrimental effects [9]; (vii) upregulation of the facilitate glucose transporter GLUT1 (SLC2A1) as well as the Na^+^-coupled concentrative glucose transporters SGLT1 (SLC5A1) and SGLT2 (SLC5A2) [10]. Collectively, these metabolic changes in cancer cells sustain “aerobic glycolysis” in the presence of sufficient oxygen and ATP (Figure 1C). As a consequence of this metabolic reprogramming, the rate of glycolysis is several-fold higher in cancer cells than in normal cells, thus generating more ATP to support their rapid proliferation and anabolic activity. Even though oxidative phosphorylation in mitochondria contributes to total ATP to some extent, a major portion, however, comes from substrate-level phosphorylation in the glycolytic pathway, enabled by the increased rate of glycolysis. Thus, in cancer cells, glucose gets converted partly into CO_2_ but mostly into lactic acid, consequently resulting in increased levels of lactate outside in the tumor microenvironment. Recent studies have shown that this extracellular lactate participates in promoting cancer cell survival and proliferation by multiple autocrine and paracrine mechanisms via the cell-surface receptor GPR81 (HCAR1 or hydroxycarboxylic acid receptor 1) [11,12]. Another important aspect of the increased rate of glycolysis in cancer cells is the elevated levels of glycolytic intermediates, which can feed into metabolic pathways that provide additional support to cancer cell proliferation. This includes: (i) glucose-6-phosphate entering into the pentose phosphate pathway to generate NADPH necessary for antioxidant machinery and pentose phosphates necessary for nucleic acid synthesis, and (ii) 3-phosphoglycerate entering into the pathway involved in the synthesis of serine and glycine, which are obligatory amino acids for the one-carbon metabolism.

## 4. Glutaminolysis and Reductive Carboxylation

Glutaminolysis is a pathway in which the carbon skeleton of glutamine is used to produce lactic acid in cancer cells; this is in some ways similar to aerobic glycolysis, where the carbon skeleton in glucose is also used to produce lactic acid. It is generally thought that lactic acid arises solely from aerobic glycolysis in cancer cells. Contrary to this widespread notion, a significant fraction of lactic acid generated in cancer cells actually comes from glutamine [13,14]. But, glutaminolysis does not stop with the production of lactic acid; carbons present in glutamine are also used in the synthesis of citrate, serine/glycine, aspartate, glutathione, fatty acids, and nucleic acids in cancer cells. Glutaminolysis involves reactions that take place in the cytoplasm as well as in the mitochondria. The first step in glutaminolysis is the conversion of glutamine into glutamate via glutaminases GLS1 and GLS2 (i.e., glutaminase I pathway) or into α-ketoglutarate via glutamine-dependent transaminases followed by ω-amidase (i.e., glutaminase II pathway) [15,16]. Of these two isoforms of glutaminase, GLS1 is known as the kidney-type and GLS2 as the liver-type. Both types are expressed at high levels in cancers, and different molecular forms of both types are localized differentially in the cytoplasm and the mitochondria [17]. Glutamine enters the mitochondria via a splice variant of the plasma membrane glutamine transporter SLC1A5 [18,19]. The subsequent fate of glutamate generated from the glutaminase I pathway varies depending on whether it is in the cytoplasm or mitochondria. In the cytoplasm, glutamate gets converted to α-ketoglutarate by reactions catalyzed by transaminases, which then could form citrate via a process called reductive carboxylation [20]. Within the mitochondria, glutamate is converted to α-ketoglutarate by glutamate dehydrogenase or glutamate-dependent transaminases, which then either enters the citric acid cycle as an intermediate in the forward direction or goes through reductive carboxylation in the reverse citric acid cycle [20]. In the former case, α-ketoglutarate provides the carbon skeleton for the synthesis of oxaloacetate, which can be used for conversion into aspartate via transamination or can leave the mitochondria in the form of malate to get into multiple metabolic pathways in the cytoplasm. Cytoplasmic glutamate can also enter mitochondria via the aspartate/glutamate carrier (AGC, SLC25A10) [21] to go through the same reactions as the glutamate generated inside the mitochondria. Similarly, citrate produced from α-ketoglutarate inside the mitochondria either through the forward passage along the citric acid cycle or through reductive carboxylation enters the cytoplasm via the citrate carrier (SLC25A1) in the inner mitochondrial membrane [21]. α-Ketoglutarate arising from the transamination reaction in the cytoplasm can enter mitochondria via the transporter OGC (oxoglutarate carrier; SLC25A11) [21] to get converted to either malate/oxaloacetate in the citric acid cycle or citrate in the reverse citric acid cycle via reductive carboxylation.

Reductive carboxylation is a metabolic pathway that mediates the conversion of α-ketoglutarate into citrate in the cytoplasm as well as inside the mitochondria. This is a pathway described and highlighted for its relevance to cancer relatively more recently [20]. Fatty acid synthesis is obligatory for any cell type with high proliferative capacity, including cancer cells. This process requires carbon sources to form the backbone in fatty acids and cholesterol, and it comes in the form of citrate offered by reductive carboxylation of α-ketoglutarate. There are two steps in the conversion of α-ketoglutarate into citrate, first the conversion of α-ketoglutarate into isocitrate and second the isomerization of isocitrate into citrate. The initial step is catalyzed by isocitrate dehydrogenase 1 (IDH1) in the cytoplasm and IDH2 inside the mitochondria. The reaction involved in both cases is essentially the reversal of the reaction catalyzed by IDH3 in the citric acid cycle. IDH3 is a NAD^+^-dependent enzyme that decarboxylates isocitrate to form α-ketoglutarate in a reaction coupled to transfer of electrons from isocitrate to NAD^+^ to generate NADH. As such, the IDH3-mediated reaction is called oxidative decarboxylation. In contrast, IDH1 in the cytoplasm and IDH2 inside the mitochondria carboxylate α-ketoglutarate using CO_2_ in a reaction coupled to the utilization of the reducing power present in NADPH. Because this reaction involves carboxylation as well as reduction, the process is called reductive carboxylation. Citrate produced inside the mitochondria gets transported into the cytoplasm via the citrate carrier SLC25A1 in the inner mitochondrial membrane [21]. Thus, citrate generated by reductive carboxylation in both compartments is available for anabolic pathways to synthesize fatty acids and cholesterol.

### 4.1. Connection of Glutaminolysis and Reductive Carboxylation to Fatty Acid/Cholesterol Synthesis

Synthesis of fatty acids and cholesterol requires the carbon source in the form of acetyl CoA and reducing power in the form of NADPH. The entire series of reactions leading to the production of fatty acids and cholesterol occurs in the cytoplasm. Malate, an intermediate in the pathway of glutaminolysis going through the forward direction of the citric acid cycle, gets out of mitochondria via SLC25A10 and/or SLC25A11 to be converted into either pyruvate by malic enzyme or oxaloacetate by malate dehydrogenase in the cytoplasm. The malic enzyme produces NADPH from NADP^+^, a significant source of reducing power for fatty acid/cholesterol synthesis; this is supplementary to the hexose monophosphate shunt pathway, which also generates NADPH. Citrate in the cytoplasm is broken down to acetyl CoA and oxaloacetate by ATP:citrate lyase; while acetyl CoA serves as the carbon backbone for the synthesis of fatty acids and cholesterol, oxaloacetate gets converted to malate to serve as the substrate for the malic enzyme, again producing NADPH to support the synthesis of fatty acids and cholesterol. Thus, glutaminolysis and reductive carboxylation offer biochemical pathways to convert the carbon skeleton in glutamine into fatty acids and cholesterol.

### 4.2. Glutamine as the Carbon Source for Lactate/Serine/Glycine Synthesis via Glutaminolysis and Reductive Carboxylation

**Figure 2:**
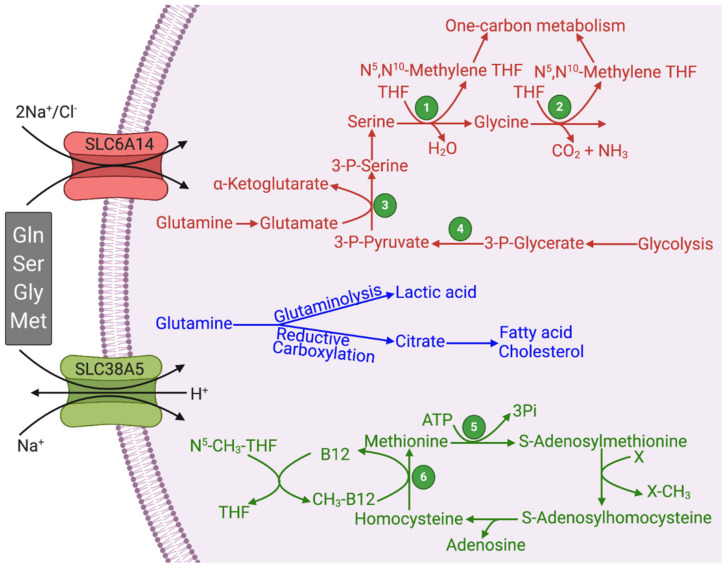
Functional features of SLC6A14 and SLC38A5 and their relevance to glutaminolysis and serine–glycine–one-carbon pathway. Figure legend 1, Serine hydroxymethyl transferase (SHMT); 2, Glycine cleavage system; 3, Phosphoserine aminotransferase (PSAT); 4, Phosphoglycerate dehydrogenase (PHGDH); 5, Methionine synthase; THF, Tetrahydrofolate.

Glutaminolysis also generates pyruvate by at least two different series of reactions. Malate produced via mitochondrial glutaminolysis can enter the cytoplasm. Cytoplasmic glutaminolysis can also generate malate via the following reactions: glutamine → glutamate → α-ketoglutarate → isocitrate → citrate → oxaloacetate → malate. Thus, cytoplasmic malate arises from glutamine both via mitochondrial glutamine breakdown and cytoplasmic glutamine breakdown. In the cytoplasm, malate is acted upon by a malic enzyme to generate pyruvate and NADPH. Pyruvate is then converted into lactate in cancer cells because of the overexpression of LDH-A. Therefore, lactic acid produced in cancer cells does not originate exclusively from aerobic glycolysis; a significant portion of lactic acid comes from glutaminolysis (Figure 2). Oxaloacetate produced from malate (malate dehydrogenase) or citrate (ATP:citrate lyase; ACLY) enters glycolysis in the reverse direction (gluconeogenesis). This requires phosphoenolpyruvate carboxykinase to make phosphoenolpyruvate, which then goes through the reversal of the reactions mediated by enolase and phosphoglycerate mutase to generate 3-phosphoglycerate. The latter is the precursor for the endogenous synthesis of serine and glycine in a series of reactions (Figure 2). The first and the rate-limiting enzyme in the series is phosphoglycerate dehydrogenase (PHGDH), which catalyzes the conversion of 3-phosphoglycerate into 3-phosphopyruvate [22]. Transaminases transfer the amino group from glutamate to 3-phosphopyruvate to make 3-phosphoserine, which then goes on to form serine. Serine serves as an important source of the one-carbon pool where the side-chain carbon in serine is transferred to tetrahydrofolate to form *N*^5^,*N*^10^-methylene tetrahydrofolate by serine hydroxymethyl transferase; glycine is the other product in the reaction. Glycine serves as an additional source of the one-carbon pool, also generating *N*^5^,*N*^10^-methylene tetrahydrofolate by the glycine cleavage enzyme complex.

### 4.3. Synthesis of Ribose-5-Phosphate and NADPH from Glutamine via 3-Phosphoglycerate

3-Phosphoglycerate, outlined in the previous section, can also go further up in the reversal of glycolysis until fructose-1,6-bisphosphate, which is converted into fructose-6-phosphate by fructose-1,6-bisphosphatase. Fructose-6-phosphate is then isomerized into glucose-6-phosphate by phosphoglucose isomerase. Hexose monophosphate shunt uses glucose-6-phosphate as the starting material and synthesizes ribose-5-phosphate and NADPH; the former can be used for the synthesis of nucleotides and the latter for the synthesis of fatty acids and cholesterol and also in cellular antioxidant machinery.

## 5. Oncometabolites: Relevance of Aerobic Glycolysis, Citric Acid Cycle, Glutaminolysis, and Reductive Carboxylation

Oncometabolite is a term used for specific bioactive compounds that are produced endogenously at low levels under normal conditions but are generated at relatively higher levels in cancer cells; the biological activities of these metabolites are related to the promotion of carcinogenesis and cancer growth. To date, four such metabolites have received significant attention, but it is almost certain that additional oncometabolites will be identified in the future. The four oncometabolites known to date are lactate [23], fumarate [24], succinate [25], and 2-hydroxyglutarate [26]. Lactate is directly related to aerobic glycolysis and glutaminolysis, fumarate and succinate to the citric acid cycle, and 2-hydroxyglutarate to the enzymes involved in reductive carboxylation. The biochemical processes that link these oncometabolites to carcinogenesis and cancer growth are the stabilization of HIF-1α via protection from proteasomal degradation, potentiation of cellular antioxidant machinery via promotion of nuclear localization of the transcription factor Nrf2, and enhancement of DNA methylation via inhibition of TET enzymes [23,24,25,26]. 

Lactate and succinate are inhibitors of prolyl hydroxylases that are involved in posttranslational modification of HIF-1α for subsequent binding to von Hippel Lindau (VHL) factor and consequent ubiquitination and proteasomal degradation. Prolyl hydroxylases use α-ketoglutarate as a cofactor [27], and lactate and succinate interfere with this reaction because of their structural similarity to the cofactor. Even though lactate and succinate have low potency as inhibitors of prolyl hydroxylases, these metabolites are produced in cancer at higher levels than in normal cells to elicit a significant inhibition of this pathway. While increased production of lactate is a hallmark of most cancers, succinate levels are elevated in certain specific types of cancers due to inactivating mutations in succinate dehydrogenase (e.g., paraganglioma). Fumarate, as an oncometabolite, was discovered in kidney cancer and is associated with inactivating mutations in fumarate hydratase (i.e., fumarase). This metabolite promotes dissociation of the Keap1/Nrf2 complex in the cytoplasm by disrupting disulfide bonds in Keap1, leading to protein-succinylation and conformational changes [28]. The resultant dissociation of the complex now allows Nrf2 to translocate to the nucleus to induce transcription of several antioxidant enzymes/transporters (e.g., heme oxygenase 1, catalytic subunit of glutamate-cysteine ligase, cystine/glutamate exchanger SLC7A11) [29,30]. The enhancement of the antioxidant machinery not only protects the cancer cells from oxidative stress but also induces chemoresistance [31,32,33,34,35]. Similar to prolyl hydroxylases, the TET enzymes involved in the demethylation of methylguanines present in the CpG islands in DNA depend on α-ketoglutarate as a cofactor [27]. Therefore, succinate is an inhibitor of this demethylation reaction also, thereby increasing DNA methylation, a known hallmark in cancer cells. In the same manner, 2-hydroxyglutarate is also a potent inhibitor of TET enzymes. Thus, succinate and 2-hydroxyglutarate elicit a profound impact on epigenetic control of gene expression in cancer cells. 2-Hydroxyglutarate is generated at high levels in cancers that have specific mutations in IDH1 and IDH2 [26]. The mutations in IDH1 and IDH2 lead to a gain of function with a new-found ability to convert α-ketoglutarate into 2-hydroxyglutarate in the presence of NADPH. The wild-type enzymes convert α-ketoglutarate into isocitrate via reductive carboxylation. In contrast, the same enzymes with the gain-of-function mutations carry out only the reduction step without the carboxylation step to generate the oncometabolite 2-hydroxyglutarate.

## 6. One-Carbon Metabolism and its Relevance to Cancer

One-carbon metabolism is fundamental to cancer cells as it is to any rapidly proliferating cell. This metabolic pathway participates in the de novo synthesis of purine and pyrimidine nucleotides and also in the control of cellular epigenetic landscape via DNA and histone methylation; the amino acids serine, glycine, and methionine serve as the primary sources of the one-carbon moiety for the reactions involved in this pathway [4,36,37,38,39]. In addition, the one-carbon metabolism mediates protein–arginine methylation with consequent changes in the biological functions of the modified proteins, which in turn contributes to modification of several biological processes related to cancer, such as protein synthesis, chemoresistance, DNA damage/repair, epigenetics, cell signaling, and tumor immunity [40,41,42]. There are three cofactors that carry out the one-carbon transfer in biochemical reactions: tetrahydrofolate (THF), vitamin B_12_, and S-adenosylmethionine (SAM). There are several one-carbon species in the one-carbon metabolism: methyl (–CH_3_), methylene (–CH_2_–), formyl (–CHO), and formimino (–CH=NH). Of these, THF is capable of participating in transfer reactions involving all four one-carbon species; as such, THF is the most versatile coenzyme in one-carbon metabolism. Vitamin B_12_ catalyzes a single reaction with one-carbon transfer where a methyl group is transferred to homocysteine to produce methionine by methionine synthase; in this reaction, the *N*^5^-methyl-THF donates the methyl group to vitamin B_12_ first, and then the methyl group is transferred from methyl-vitamin B_12_ to homocysteine (Figure 2). This is the only reaction in mammalian systems where a methyl transfer reaction occurs without SAM. In all other methyl transfer reactions, SAM is the coenzyme that serves as the methyl donor. The principal sources of one-carbon species for these one-carbon transfer reactions are serine and glycine. With these donors of the one-carbon moiety, THF gets converted to *N*^5^, *N*^10^-methylene THF, which can be converted to other one-carbon species attached to THF. Methionine serves as the donor of the methyl group in the form of SAM. When the methyl group is transferred from SAM to an acceptor, the resultant S-adenosyl homocysteine gets converted to homocysteine subsequently, which is recycled back to methionine using the methyl group in *N*^5^-methyl-THF with the intermediate involvement of vitamin B_12_. There is also an alternative pathway for salvage of methionine in which glutamine serves as the donor of the amino group in a transamination reaction; here, the α-amino group in glutamine is transferred to 2-keto-4-methylthiobutyrate to generate methionine from 5′-methylthioadenosine [43,44,45].

The THF-associated one-carbon transfer is obligatory for the synthesis of purine and pyrimidine nucleotides. *N*^10^-formyl-THF participates in the assemblage of purine and pyrimidine bases; *N*^5^, *N*^10^-methylene-THF participates in the conversion of dUMP into TMP. SAM serves as the methyl donor for DNA methylation mediated by DNA methyltransferases and methylation of lysine residues in histones and arginine residues in non-histone proteins.

## 7. SLC6A14 and SLC38A5 and Their Relevance to Cancer

Cancer cells exhibit increased demands for all amino acids, essential as well as non-essential. Since the essential amino acids cannot be generated de novo, these have to be obtained solely from extracellular sources. In contrast, the non-essential amino acids can be produced endogenously in cells from other precursors; nonetheless, cancer cells rely on extracellular sources to a significant extent even for these amino acids because their synthetic capacity does not meet the highly increased demands. Therefore, cancer cells upregulate specific amino acid transporters in their plasma membrane to mediate the entry of essential and non-essential amino acids from blood. To date, four distinct amino acid transporters have received increasing attention for their role in the promotion of cancer: SLC1A5 (also known as ASCT2), SLC7A5 (also known as LAT1), SLC7A11 (also known as x_c_^−^), and SLC6A14 (also known as ATB^0,+^) [46,47,48,49,50]. SLC38A5 (also known as SN2) [51,52] should also be added to this elite list prompted by the findings that it is a target for the c-Myc oncogene [53], but relatively much less is known on this transporter in cancer compared to the other four transporters. Among these five amino acid transporters that have received attention with regard to their biological relevance to cancer, SLC6A14 and SLC38A5 stand out in terms of their functional features that are ideal for the promotion of cancer (Table 1).

SLC6A14 and SLC38A5 mediate the transfer of their amino acid substrates in one direction, mostly into the cells in the influx mode (Figure 2). SLC6A14 is also known as ATB^0,+^, referring to its identity with the amino acid transport system B^0,+^ (B stands for “broad-specific”; uppercase letter stands for Na^+^-dependence; 0,+ in the superscript stands for the ability of the transporter to accept neutral and cationic amino acids as substrates). It is energized by three driving forces: a Na^+^ gradient, a Cl^−^ gradient, and membrane potential [54,55,56]. Therefore, this transporter primarily mediates the influx of its substrates into cells because of the high magnitude of the combined driving forces. SLC38A5 is also known as SN2 (i.e., the second isoform of the amino acid transport system N that is capable of accepting the amino acids glutamine, asparagine, and histidine, which contain nitrogen in the side chain; N stands for nitrogen in the side chain) and SNAT5 (sodium-coupled neutral amino acid transporter 5). It is also driven by a Na^+^ gradient, but interestingly it is an electroneutral transporter with no involvement of membrane potential. This is because of the coupling of the transport process with a transmembrane H^+^ gradient in which the transfer of Na^+^ and the amino acid substrate in one direction is functionally coupled to the transfer of H^+^ in the opposite direction [51,52,57]. As SLC38A5 accepts only neutral amino acids as its substrates, the transport process is electroneutral. Based on this operational mechanism, the transfer of amino acids and Na^+^ into cells via SLC38A5 leads to the removal of H^+^ from the cells, thus resulting in intracellular alkalinization [52]. Stated differently, SLC38A5 functions as an amino acid-dependent Na^+^/H^+^ exchanger. Since the transmembrane Na^+^ gradient is quantitatively the principal driving force for SLC38A5 under normal physiologic conditions, this transporter could also mediate the efflux of glutamine from the cells if the cells express high levels of glutamine synthetase, thus causing a concentration gradient for the amino acid across the plasma membrane in the outward direction. This is the case for the function of this transporter in astrocytes in the brain and Muller cells in the retina where SLC38A5 mediates the release of glutamine from the cells with subsequent uptake of glutamine by neurons; this is a part of the glutamine–glutamate cycle that operates between astrocytes/Muller cells and neurons [58,59]. However, the functioning of this transporter in the glutamine efflux mode is unique to astrocytes/Muller cells. This transporter is expected to function solely in the influx mode in cancer cells because of the avid utilization of glutamine by these cells, thus creating a concentration gradient for the amino acid across the plasma membrane that facilitates influx rather than efflux. In contrast to SLC6A14 and SLC38A5, the other three transporters related to cancer are all amino acid exchangers, meaning that the influx of one amino acid substrate into cells is coupled to the efflux of another amino acid substrate out of the cells [48]. Consequently, these three transporters are not ideal to meet the increased demands for amino acids in cancer cells because when the cells acquire one given amino acid via these transporters, they lose some other amino acid.

The substrate selectivity of SLC6A14 and SLC38A5 bears a key connection to glutaminolysis and serine–glycine–one-carbon pathway. Both transporters transport glutamine, serine, glycine, and methionine (Figure 2; Table 1). Some functional features do distinguish the two transporters. SLC6A14 transports 18 of the 20 proteinogenic amino acids, including all essential amino acids; as leucine is a potent activator of oncogenic mTOR signaling, the functional activity of this transporter is coupled to mTOR activation. Both of these features are essential for cancer cells to support their growth and proliferation. In contrast, the substrate selectivity of SLC38A5 is relatively restricted. This transporter can mediate the uptake of only glutamine, asparagine, histidine, serine, glycine, and methionine; this list of substrates contains all the four amino acids related to glutaminolysis and one-carbon metabolism. However, SLC38A5 exhibits a functional feature that is not seen with SLC6A14. The transport function of SLC38A5 is coupled to H^+^ efflux. As cancer cells generate massive amounts of lactic acid, there is an absolute need for these cells to prevent intracellular acidification, and SLC38A5 contributes to this process. The resultant intracellular alkalinization promotes the entry of the cells into the DNA-synthesis phase of the cell cycle and consequently accelerates cell proliferation [60,61]. It has been documented that SLC38A5 causes an increase in pH in the immediate vicinity of the plasma membrane on the cytoplasmic side of the cell in the presence of its amino acid substrates [52]. This effect is expected to promote another nutrient delivery system, known as macropinocytosis, in cancer cells. The NHE1 isoform (SLC9A1) of the Na^+^/H^+^ exchanger is known for its mitogenic potential with its role in the prevention of intracellular acidification as well as in the promotion of macropinocytosis [62]; inhibitors of NHE1 are widely used to block macropinocytosis [63]. The alkaline pH on the cytoplasmic surface of the plasma membrane influences the phosphorylation and activity of cytoskeletal scaffold proteins to initiate and promote macropinocytosis, a non-selective mode of uptake of nutrients from the extracellular milieu by “drinking” minute droplets of extracellular fluid [64,65,66,67]. As mentioned earlier, SLC38A5 functions as an amino acid-dependent Na^+^/H^+^ exchanger. Therefore, this transporter is expected to promote macropinocytosis in the presence of its amino acid substrates, such as glutamine, serine, glycine, asparagine, histidine, and methionine. It has already been documented that increased activity of macropinocytosis is a hallmark of certain types of solid tumors, particularly tumors associated with KRAS mutations [66,67]. 

## 8. Potential Functional Coupling between SLC6A14/SLC38A5 with Other Transporters

Since glutamine is a good substrate for SLC6A14 and SLC38A5, the Na^+^-coupled concentrative influx of these amino acids into cancer cells is likely to activate the heterodimeric amino acid transporter known as cystine/glutamate exchanger (SLC7A11/4F2hc). This exchanger mediates the influx of the disulfide amino acid cystine into cells coupled to the efflux of the anionic amino acid glutamate. Glutamine concentrated inside the cells via SLC6A14 and SLC38A5 is expected to generate glutamate either by glutaminases in the glutaminase I pathway or by glutamine-dependent transaminases in the glutaminase II pathway. This glutamate would provide the exchange amino acid to facilitate the influx of cystine via SLC7A11/4F2hc. This would represent an important biological process for the cancer cells because cystine entering the cells will be converted into cysteine within the cells, which along with glutamate and glycine, will form glutathione, thus enhancing the antioxidant machinery in cancer cells. In a similar manner, it is also possible that SLC38A5 and the H^+^-coupled lactate transporter MCT1 (SLC16A1) are functionally coupled in cancer cells via H^+^. The transport function of SLC38A5 involves the influx of Na^+^ and amino acid substrates into cells coupled to the efflux of H^+^. It is known that lactate generated in hypoxic cancer cells that are located far away from blood supply is used as an energy substrate by oxygenated cancer cells that lie in close proximity to blood vessels; this process involves the entry of lactate via MCT1 into lactate-using cells coupled to H^+^ influx. This H^+^ would serve as a substrate for SLC38A5 to increase the transporter’s ability to mediate the influx of amino acids into the cells. The vice versa is also possible in which the H^+^ efflux mediated by SLC38A5 fuels the entry of lactate/H^+^ via MCT1. Such a mutual stimulation of the two transporters would be an ideal phenomenon to support cancer cell survival and proliferation. Nonetheless, the aforementioned ideas on the functional coupling between SLC6A14/SLC38A5 and SLC7A11/4F2hc and between SLC38A5 and MCT1 remain only speculative at this time and have to wait for experimental supporting evidence. 

## 9. Upregulation of SLC6A14 and SLC38A5 in Cancer Cells and Signaling Mechanisms Involved in the Process

Several reports have shown marked upregulation of SLC6A14 in many types of solid tumors, including colon cancer [68,69], cervical cancer [70,71], estrogen receptor-positive breast cancer [72,73,74], and pancreatic cancer [75,76,77,78,79,80]. The role of SLC6A14 as a potential biomarker for survival and prognosis has been validated, particularly in pancreatic ductal adenocarcinoma, with higher expression levels in tumors correlating with reduced survival [75,76,77,78,79,80]. These findings strongly suggest that SLC6A14 functions as a tumor promoter. The signaling mechanisms responsible for the upregulation of this transporter in cancer are just beginning to be understood. Estrogen induces the expression of the transporter [73], thus providing a molecular mechanism for the upregulation of the transporter, specifically in estrogen receptor-positive breast cancer. The involvement of Wnt signaling in the upregulation of SLC6A14 has been described recently [69]. Signaling pathways initiated by Wnt ligands are known to be associated with carcinogenesis [81,82]. Therefore, it seems that the upregulation of SLC6A14 with its tumor-promoting capability represents at least one of the molecular mechanisms by which estrogen signaling and Wnt signaling drive carcinogenesis and tumor growth.

Relatively much less is known on the expression and function of SLC38A5 in cancer. A recent report has indicated a significant correlation between the expression level of this transporter in cancers and resistance to cisplatin efficacy [83]. The Cancer Genome Atlas (TCGA) database provides evidence for the upregulation of SLC38A5 at least in pancreatic cancer and an inverse correlation between the expression levels of the transporter and survival in patients with this cancer. Despite the paucity of data on SLC38A5 in cancer, there is convincing evidence for the role of this transporter in promoting cell proliferation. In the intestinal tract, this transporter is expressed specifically in crypt cells where it provides the cells with glutamine and other amino acids [84]; these cells normally exhibit a high proliferative capacity. The expression of the transporter is induced under pathological conditions associated with increased expansion of the intestinal epithelial cells [85,86,87]. Single-cell transcriptomic profiling has shown SLC38A5 as a characteristic gene for the progenitors of glucagon-secreting α-cells [88]. Amino acid delivery via this transporter drives α-cell hyperplasia [89,90], and the elevation of amino acids in blood induces a subpopulation of α-cells to form pancreatic neuroendocrine tumors [91]. Interestingly, similar to SLC6A14, the expression of SLC38A5 is also under the control of the Wnt signaling pathway [92,93,94], thus suggesting a probable connection between this transporter and cancer. 

## 10. SLC6A14 and SLC38A5 as Actionable Drug Targets for Cancer Therapy

SLC6A14 and SLC38A5 are cell-surface proteins that support amino acid nutrition in cancer cells. The amino acids supplied by these transporters serve not only as the building blocks for protein synthesis but also function as signaling molecules (e.g., mTOR activation) and provide substrates for the cancer-cell-specific metabolic pathways, such as glutaminolysis and serine–glycine–one-carbon pathway. As such, SLC6A14 and SLC38A5 drive cancer cell proliferation and tumor growth in specific types of solid tumors. In theory, if small molecules can be developed that have high affinity and high selectivity to inhibit the transport function of these transporters, such inhibitors could potentially have efficacy as anticancer drugs. This idea has already been validated with α-methyl-L-tryptophan as a fairly selective inhibitor of SLC6A14 [72]. It is important to note that α-methyl-L-tryptophan is not a transportable substrate for SLC6A14; it binds to the substrate-binding site of the transporter and blocks the transport function without itself being transported into the cells. As such, α-methyl-L-tryptophan is a blocker of the transporter rather than a competitive inhibitor. The IC_50_ value for the L-enantiomer to block the transport function of SLC6A14 is ~10 μM. With this small molecule, published reports have demonstrated the therapeutic utility of SLC6A14 blockade in the treatment of multiple tumor types that are associated with upregulation of this transporter: breast cancer [72,73,74], pancreatic cancer [76,95], and colon cancer [69]. 

There have been some studies describing selective inhibitors of SLC38A5 [96]; the best inhibitor in terms of selectivity is glutamate-γ-hydroxamate. It is a competitive inhibitor of the transporter, but it is not known whether the compound is actually transported into cells via the transporter. Furthermore, the inhibitor shows low affinity with an IC_50_ value of ~0.6 mM. Even though selective high-affinity inhibitors/blockers for SLC38A5 are not yet available, the experience with α-methyl-L-tryptophan and SLC6A14 indicates that a similar approach would also work for SLC38A5-positive tumors as well. Another feasible approach is to generate monoclonals specific to extracellular epitopes of these transporters that could be used to block the function of these cell-surface proteins. Even though it is widely acknowledged that amino acid metabolism comprising of numerous biochemical pathways is obligatory for cancer cell survival and that amino acid transporters play an essential role in supplying the amino acid substrates to feed into these pathways, the possibility of exploiting these transporter proteins as actionable drug targets for cancer therapy is just beginning to be recognized. If successful, this would represent a novel, hitherto unexplored, therapeutic approach for the treatment of cancer.

## 11. Conclusions

Cancer cells are “addicted” to glutamine; this amino acid plays a multitude of biological functions, all of which are critical for cell proliferation. In a similar manner, the one-carbon metabolism is also obligatory for cell proliferation because of its role in various cellular processes; the reactions involved in this metabolism cannot occur without serine, glycine, and methionine as the suppliers of the one-carbon moiety. Therefore, the amino acid transporters that have the ability to provide glutamine, serine, glycine, and methionine to cells have the potential to drive cancer cell proliferation. SLC6A14 and SLC38A5 represent two such transporters with their unique amino acid substrate profiles and functional features that have been shown to be connected to cancer. These two transporters are upregulated in cancer, and pharmacological blockade of their transport function has potential for cancer therapy. As such, small molecule inhibitors/blockers or monoclonal antibodies that have the ability to interfere with the function of SLC6A14 and SLC38A5 might represent a novel class of anticancer drugs.

## Figures and Tables

**Table 1:** Functional features of SLC6A14 and SLC38A5 that distinguish these two transporters from other amino acid transporters known to be upregulated in cancer.

Feature	SLC6A14	SLC38A5	SLC7A5	SLC1A5	SLC7A11
Transport of Glutamine	+	+	+	+	-
Transport of all essential AA	+	−	+	−	−
Transport of mTOR activator Leu	+	−	+	−	−
Transport of Ser and Gly	+	+	−	+	−
Transport of Methionine	+	+	+	−	−
Transport of Cystine	−	−	−	−	+
Energy from membrane potential	+	+	−	−	−
Uniport of AA into cells	+	+	−	−	−
Mitogenic alkalinization	−	+	−	−	−
Macropinocytosis	−	+	−	−	−

## Data Availability

All data pertaining to this review are included in this manuscript.
